# Effect of Basal Metabolic Rate on Cancer: A Mendelian Randomization Study

**DOI:** 10.3389/fgene.2021.735541

**Published:** 2021-09-09

**Authors:** Jack C. M. Ng, C. Mary Schooling

**Affiliations:** ^1^School of Public Health, Li Ka Shing Faculty of Medicine, The University of Hong Kong, Hong Kong Special Administrative Region, China; ^2^Department of Environmental, Occupational, and Geospatial Health Sciences, Graduate School of Public Health and Health Policy, The City University of New York, New York, NY, United States

**Keywords:** cancer, Mendelian randomization, basal metabolic rate, metabolism, evolutionary biology

## Abstract

**Background:** Basal metabolic rate is associated with cancer, but these observations are open to confounding. Limited evidence from Mendelian randomization studies exists, with inconclusive results. Moreover, whether basal metabolic rate has a similar role in cancer for men and women independent of insulin-like growth factor 1 increasing cancer risk has not been investigated.

**Methods:** We conducted a two-sample Mendelian randomization study using summary data from the UK Biobank to estimate the causal effect of basal metabolic rate on cancer. Overall and sex-specific analysis and multiple sensitivity analyses were performed including multivariable Mendelian randomization to control for insulin-like growth factor 1.

**Results:** We obtained 782 genetic variants strongly (*p*-value < 5 × 10^–8^) and independently (*r*^2^ < 0.01) predicting basal metabolic rate. Genetically predicted higher basal metabolic rate was associated with an increase in cancer risk overall (odds ratio, 1.06; 95% confidence interval, 1.02–1.10) with similar estimates by sex (odds ratio for men, 1.07; 95% confidence interval, 1.002–1.14; odds ratio for women, 1.06; 95% confidence interval, 0.995–1.12). Sensitivity analyses including adjustment for insulin-like growth factor 1 showed directionally consistent results.

**Conclusion:** Higher basal metabolic rate might increase cancer risk. Basal metabolic rate as a potential modifiable target of cancer prevention warrants further study.

## Introduction

Cancer mortality ranks second among causes of death worldwide (World Health Organization, 2018) despite significant advances in cancer treatment, provision of screening programs, and reduction of specific environmental hazards, such as smoking, asbestos, and cancer-provoking infections. Owing to its complex, varied, and multifactorial nature, prevention involves focusing on factors driving specific cancers as well as consideration of more broad factors, such as viral infections, diet, and lifestyle (Lampe, 2020), that generally increase vulnerability to cancer. To inform this wider perspective, concepts from evolutionary biology are increasingly being applied to develop novel prevention and treatment strategies (Aktipis and Nesse, 2013; Boddy et al., 2015; Schooling, 2017).

Evolutionary biology suggests reproductive success may take precedence over health and longevity (Wells et al., 2017). Faster growth and earlier maturation favor reproductive success (Aktipis and Nesse, 2013; Wells et al., 2017), possibly with different trade-offs by sex. Increasing evidence suggests that growth hormone and associated factors, such as insulin-like growth factor 1 (IGF1), which regulates cell growth, play a role in cancer (Swanson and Dantzer, 2014; Murphy et al., 2020a,b). Correspondingly, most Mendelian randomization (MR) studies have shown a positive association of IGF1 with different types of cancer (Cornish et al., 2020; Larsson et al., 2020; Murphy et al., 2020a,b; Watts et al., 2021). Interventions to reduce IGF1 are under investigation (Werner and Laron, 2020). Basal metabolic rate (BMR) is a related potential target of intervention. Observationally, BMR is associated with cancer (Kliemann et al., 2020). Observational studies are open to confounding, which may compromise internal validity. No randomized controlled trial has been performed to examine the effect of BMR on cancer. MR is a widely used alternative approach taking the advantage of Mendel’s second law, where genetic variants (GVs) randomly assort at conception are used to obtain unconfounded estimates (Smith and Ebrahim, 2003). MR studies on this topic are limited. One MR study reported that BMR was suggestively associated with colorectal cancer (Cornish et al., 2020). Another reported a null association with glioma (Saunders et al., 2020).

In the current study, we conducted an MR analysis to investigate the effect of BMR on cancer and neoplasms overall and by sex using summary data from the UK Biobank. We also conducted exploratory analysis for specific cancers, where possible. Given the interrelation between IGF1 and BMR (Swanson and Dantzer, 2014), we also examined whether any effects of BMR were independent of IGF1 using multivariable MR.

## Materials and Methods

We performed a univariable and multivariable MR study of the association of BMR with cancer as the primary outcome and neoplasm as secondary outcome using a two-sample summary data approach, where we applied genetic predictors of the exposure, BMR, to genome-wide association studies (GWASs) of the outcomes. For exploratory purpose, we also considered several site-specific cancers based on the International Classification of Diseases-10 codes. As an instrumental variable analysis, MR assumes strong genetic predictors of the exposure, no confounding of the instruments or exposure on outcome, and that the instruments only affect the outcome *via* effects on the exposure.

### Genetic Predictors of Basal Metabolic Rate

Genetic predictors of BMR were obtained from the summary statistics of the UK Biobank GWAS provided by the Neale Lab (2018). The UK Biobank is a population-based, prospective cohort study for different health outcomes that aimed to recruit >500,000 individuals aged 40 to 69 years during 2006 to 2010 (Sudlow et al., 2015). BMR (KJ) was inverse-ranked normalized (phenotype code, 23105_irnt), i.e., effect sizes, for all analyses. Neale Lab (2018) analyzed the genetic associations using multivariable linear regression in 361,194 people (54% women) of white British ancestry with adjustment for age, age × age, inferred sex, age × inferred sex, age × age × inferred sex, and the first 20 principal components in overall analysis and age, age × age, and the first 20 principal components in sex-specific analysis.

We excluded GVs that are non-biallelic, rare (minor allele frequency <0.01), not in Hardy-Weinberg equilibrium (*p*-value for Chi-squared test <0.05), and from sex chromosomes in overall-sex analysis. We did not exclude GVs from sex chromosomes in sex-specific analysis. Only GVs strongly (*p*-value < 5 × 10^–8^) and independently (*r*^2^ < 0.01) predicting BMR were retained. We used the “clump_data” function in the “TwoSampleMR” R package to identify independent GVs (Hemani et al., 2018).

### Genetic Associations With Cancer and Neoplasm

Genetic associations with cancer and neoplasm obtained using multivariable linear regression were extracted from the same data source as for BMR (Neale Lab, 2018). The analyzed sample consisted of 28,509 cases of cancer diagnosed by doctor (phenotype code, 2453) and 70,178 cases of neoplasm (phenotype code, II_NEOPLASM).

### Genetic Associations With Site-Specific Cancers

Genetic associations with several site-specific cancers, namely (i) cancer of lip, oral cavity, and pharynx, (ii) cancer of digestive organs, (iii) cancer of respiratory system and intrathoracic organs, (iv) skin cancer, (v) cancer of mesothelium and soft tissue, (vi) cancer of urinary organs, (vii) cancer of eye, brain, and central nervous system, (viii) cancer of endocrine gland, (ix) cancer of primary lymphoid and hematopoietic tissue, (x) cancer of genital organs in men, (xi) prostate cancer, (xii) breast cancer in women, and (xiii) cancer of genital cancers in women were obtained from the same data source as for BMR (Neale Lab, 2018). The Prostate Cancer Association Group to Investigate Cancer Associated Alterations in the Genome (PRACTICAL) Consortium (Schumacher et al., 2018) and the Breast Cancer Association Consortium (BCAC) (Zhang et al., 2020) were used for replication. The study details for each site-specific cancer are given in Supplementary Table 1.

### Sex-Specific Analysis

We carried out sex-specific analysis for primary and secondary outcomes as well as cancer of genital organs in men and women, prostate cancer, and breast cancer in women using sex-specific genetic predictors of BMR and IGF1. We assessed sex differences for primary and secondary outcomes using a *z*-test (Paternoster et al., 1998).

### Assessment of Confounding

To assess potential confounding, we checked whether the GVs predicting BMR were also related to five potential confounders of the BMR-cancer association, namely Townsend index (phenotype code, 189), age at completion of full-time education (phenotype code, 845), number of days per week walked for 10 + min (phenotype code, 864), current smoking (phenotype code, 1239), and alcohol intake frequency (phenotype code, 1558) using summary statistics from the same data source (Neale Lab, 2018), at Bonferroni-corrected *p*-value, i.e., 0.05/(number of GVs × number of traits).

### Statistical Analysis

We aligned the effect of a GV across the exposure and outcome datasets based on the effect allele and reference allele and checked against the allele frequency. For palindromic GVs, i.e., GVs with allele pair of A/T or C/G, we discarded those with minor allele frequency ≥0.45 (close to 0.50) if the strand information was not given and the exposure and outcome originated from different data sources. Alternatively, we discarded all palindromic GVs, if both minor allele frequency and strand information were not available. The GV-specific Wald estimate was calculated as the ratio of the estimate of GV-outcome association to that of GV-exposure association (Wald, 1940; Palmer et al., 2011). The standard error of the Wald estimate was approximated from Fieller’s theorem (Fieller, 1954; Burgess et al., 2015), which effectively assumes “NO Measurement Error (NOME)” for the exposure (Bowden et al., 2016b).

We assessed the strength of instruments from the F-statistic using an approximation, i.e., dividing squared beta by squared standard error of the association of GV with exposure (Bowden et al., 2016b), with a value <10 indicating a weak instrument (Bowden et al., 2016b).

In the primary MR analysis, the GV-specific Wald estimates per 1 unit of effect size change in genetically predicted BMR were meta-analyzed using inverse-variance weighted with multiplicative random effects, which assumes balanced pleiotropy (Bowden et al., 2016b; Hemani et al., 2018). Other MR methods used as sensitivity analysis included weighted median and MR-Egger, using the “MendelianRandomization” R package (Yavorska and Burgess, 2017), as well as MR-pleiotropy residual sum and outlier (MR-PRESSO) (with 10,000 simulations performed), using the “MR-PRESSO” R package (Verbanck et al., 2018). The weighted median gives a consistent estimate when at least 50% of the weight is from valid GVs (Bowden et al., 2016a). MR-Egger gives a consistent estimate under the “Instrument Strength Independent of Direct Effect” assumption (Bowden et al., 2016b; Burgess and Thompson, 2017), for example, assuming the instrument does not affect an exposure-outcome confounder or affect survival when confounders of survival and outcome exist (Schooling et al., 2021). The *I*^2^_GX_ statistic in MR-Egger reflects the instrument strength and the extent of violation of the “NOME” assumption (Bowden et al., 2016b). A low value indicates possible bias of the estimate towards null. A non-zero MR-Egger intercept indicates the presence of directional pleiotropy, which invalidates the inverse-variance-weighted estimate (Burgess and Thompson, 2017). MR-PRESSO identifies outliers as a statistical means to detect directional pleiotropy and correct the estimate by removing the outliers (Verbanck et al., 2018). In overall analysis on cancer and neoplasm, we also applied the contamination mixture method using the “MendelianRandomization” R package (Yavorska and Burgess, 2017) because simulation suggests it provides the most reliable MR estimate (Slob and Burgess, 2020).

To address the possibility of BMR affecting cancer via IGF1 rather than BMR, i.e., horizontal pleiotropy, given its correlation with BMR (Swanson and Dantzer, 2014), we also adjusted for IGF1 (nmol/L) that was inverse-rank normalized (phenotype code, 30770_irnt) by multivariable MR, using summary statistics from the same data source as for BMR (Neale Lab, 2018). We combined genetic predictors for BMR and IGF1 and dropped any correlated GVs. We assessed the instrument strength from the conditional F-statistic using the “MVMR” R package (Sanderson et al., 2019). We obtained the multivariable inverse-variance-weighted estimates with multiplicative random effects in primary analysis and multivariable MR-Egger estimates as a sensitivity analysis using the “MendelianRandomization” R package (Yavorska and Burgess, 2017). The MR-Egger intercept test is sensitive to the orientation of GVs (Burgess and Thompson, 2017) and the orientation may affect the estimates in multivariable MR-Egger (Rees et al., 2017). As BMR was the exposure of interest, we performed analysis by orientating the GVs with respect to the risk-increasing allele for BMR. The multivariable MR estimates represent the direct effect of the exposure on the outcome (Sanderson et al., 2019). We also assessed the heterogeneity of multivariable estimates by the modified Cochran’s Q statistic, which reflects the extent of residual horizontal pleiotropy, using the “MVMR” R package (Sanderson et al., 2019). Given, we used summary statistics for the exposures from the same study without accounting for their covariance, the conditional F-statistic likely represents a lower bound and the modified Cochran’s *Q* statistic likely represents an upper bound.

We repeated all MR analyses by excluding GVs associated with potential confounders at Bonferroni-corrected *p*-value.

MR estimates for cancer, neoplasm, and site-specific cancers in the UK Biobank were presented as odds ratio (OR) after converting the probability to log odds by an approximation as calculated by [(*k* + *b*)/(1 – *k* – *b*)] × [(1 – *k*)/*k*], where *k* is the proportion of cases in the sample and *b* is the MR estimate in probability (Lloyd-Jones et al., 2018).

Power calculations were performed for overall analysis using the approximation that the sample size of the outcome for an MR study is the sample size for exposure on outcome divided by the proportion of variance for instruments on exposure (Freeman et al., 2013), using an online calculator (Anonymous, 2021). The proportion of variance in BMR explained by the each GV was calculated by an approximation as beta^2^ × 2 × minor allele frequency × (1 – minor allele frequency), where beta is the effect size of the genetic association of GV with BMR and minor allele frequency is represented by effect allele frequency (Del Greco et al., 2017). An overall proportion of variance was obtained by summing GV-specific ones.

All statistical analyses were conducted using R (version 3.6.3, The R Foundation for Statistical Computing Platform, Vienna, Austria).

### Ethics Approval

The current MR study used only publicly available summary data, with no original data collected. Ethical approval for the UK Biobank and the GWASs, including written informed consent from individual participants, can be found in the original publications.

## Results

### Genetic Predictors of Basal Metabolic Rate and Insulin-Like Growth Factor 1

We obtained 782 GVs strongly and independently predicting BMR (Supplementary Table 2), of which all were available for cancer and neoplasm since they originate from the same GWAS. Their mean F-statistic was 60 (range, 30 to 679) (Supplementary Table 3). Supplementary Table 4 shows the GVs predicting BMR associated with potential confounders. The GVs explained approximately 5.6% of the variance in BMR. The study has a power of >80% to detect an OR of 1.08 for cancer and an OR of 1.06 for neoplasm per 1 unit of effect size change in genetically predicted BMR.

We obtained 604 GVs strongly and independently predicting IGF1 (Supplementary Table 5), which also all were available for cancer and neoplasm. Their mean F-statistic was 77 (range, 30 to 1997) (Supplementary Table 4). Supplementary Table 6 shows the GVs predicting IGF1 associated with potential confounders.

Supplementary Tables 2, 5 show the associations of BMR and IGF1, respectively, with site-specific cancers.

The conditional F-statistic for the multivariable analysis of the association of BMR and IGF1 with cancer and neoplasm was 39 for BMR and 40 for IGF1 (Supplementary Table 7).

### Genetic Associations of Basal Metabolic Rate With Cancer

BMR was positively associated with cancer (Table 1). Other MR methods and inclusion/exclusion of GVs associated with potential confounders showed directionally similar results. No MR-Egger intercept tests showed evidence of directional pleiotropy. BMR was positively associated with cancer using the contamination mixture method (odds ratio, 1.13; 95% confidence interval, 1.09 to 1.20). Exclusion of GVs associated with potential confounders showed directionally similar results.

**TABLE 1:** Univariable Mendelian randomization association of basal metabolic rate with cancer.

**Sex**	**Selection of GVs**	**No. of GVs**	**MR method**	**Odds ratio**	**95% CI LL**	**95% CI UL**	***p*-Value**	**Cochran’s *Q***		**MR-Egger**			**Outlier detected by MR-PRESSO**	***p*-Value for sex difference**
								**Statistic**	***p*-Value**	**Intercept**	**Intercept *p*-Value**	***I*^2^_GX_**		
Overall	*p*-Value < 5 × 10^–8^	782	IVW (random)	1.06	1.02	1.10	0.006	923.082	0.0003	–	–	–	–	
		782	WM	1.05	0.99	1.12	0.102	–	–	–	–	–	–	
		782	MR-Egger	1.01	0.91	1.13	0.798	922.191	0.0003	6.57E-05	0.385	0.834	–	
		781	MR-PRESSO (corrected)	1.07	1.03	1.11	0.001	–	–	–	–	–	rs56094641	
	*p*-Value < 5 × 10^–8^ and exclusion of GVs associated with potential confounders	750	IVW (random)	1.07	1.02	1.11	0.003	859.463	0.003	–	–	–	–	
		750	WM	1.07	1.01	1.14	0.033	–	–	–	–	–	–	
		750	MR-Egger	1.05	0.93	1.17	0.400	859.368	0.0028	2.27E-05	0.774	0.795	–	
		750	MR-PRESSO^a^	1.07	1.02	1.11	0.003	–	–	–	–	–	–	
Men	*p*-Value < 5 × 10^–8^	301	IVW (random)	1.07	1.002	1.14	0.044	346.633	0.033	–	–	–	–	0.840
		301	WM	0.99	0.89	1.09	0.827	–	–	–	–	–	–	0.471
		301	MR-Egger	1.07	0.88	1.26	0.462	346.633	0.030	-2.00E-06	0.991	0.763	–	0.162
		300	MR-PRESSO (corrected)	1.06	0.997	1.13	0.061	–	–	–	–	–	rs1879442	0.380
	*p*-Value < 5 × 10^–8^ and exclusion of GVs associated with potential confounders	281	IVW (random)	1.08	1.01	1.16	0.027	328.480	0.024	–	–	–	–	0.851
		281	WM	0.999	0.89	1.10	0.979	–	–	–	–	–	–	0.541
		281	MR-Egger	1.13	0.93	1.35	0.204	328.158	0.023	-9.54E-05	0.601	0.693	–	0.178
		281	MR-PRESSO^a^	1.08	1.01	1.16	0.028	–	–	–	–	–	–	0.851
Women	*p*-Value < 5 × 10-8	304	IVW (random)	1.06	0.995	1.12	0.069	405.209	7.65E-05	–	–	–	–	
		304	WM	1.03	0.95	1.12	0.453	–	–	–	–	–	–	
		304	MR-Egger	0.89	0.73	1.07	0.231	400.115	0.0001	0.0004	0.0499	0.762	–	
		303	MR-PRESSO (corrected)	1.08	1.02	1.14	0.010	–	–	–	–	–	rs56094641	
	*p*-Value < 5 × 10^–8^ and exclusion of GVs associated with potential confounders	280	IVW (random)	1.07	1.003	1.13	0.041	336.074	0.011	–	–	–	–	
		280	WM	1.04	0.95	1.13	0.440	–	–	–	–	–	–	
		280	MR-Egger	0.93	0.75	1.12	0.474	333.389	0.013	0.0003	0.135	0.686	–	
		280	MR-PRESSO^a^	1.07	1.002	1.13	0.042	–	–	–	–	–	–	

*BMR, basal metabolic rate; CI, confidence interval; GV, genetic variant; IVW, inverse-variance weighted; LL, lower limit; MR, Mendelian randomization; MR-PRESSO, Mendelian randomization-pleiotropy residual sum and outlier; SE, standard error; UL, upper limit; WM, weighted median.*

*^a^No outlier was detected.*

In sex-specific analysis, estimates were similar to the overall estimates and by sex (Table 1). Other MR methods with inclusion/exclusion of GVs associated with potential confounders generally showed a positive association. Only the MR-Egger intercept test for the analysis without exclusion of GVs associated with potential confounders in women showed evidence of directional pleiotropy.

In multivariable MR, BMR was positively associated with cancer after adjusting for IGF1 overall and in women but not in men (Table 2). MR-Egger and inclusion/exclusion of GVs associated with potential confounders showed directionally similar results. No MR-Egger intercept showed evidence of directional pleiotropy.

**TABLE 2:** Multivariable Mendelian randomization association of basal metabolic rate with cancer adjusted for insulin-like growth factor 1.

**Sex**	**Selection of GVs**	**No. of GVs**	**MR method**	**Odds ratio**	**95% CI LL**	**95% CI UL**	***p*-Value**	**Cochran’s *Q***		**MR-Egger**		***p*-Value for sex difference**
								**Statistic**	***p*-Value**	**Intercept**	**Intercept *p*-value**	
Overall	*p*-Value < 5 × 10^–8^	1,152	IVW (random)	1.07	1.02	1.11	0.003	1,350.644	3.50E-05	–	–	
		1,152	MR-Egger	1.03	0.96	1.11	0.423	1,349.144	3.62E-05	4.83E-05	0.258	
	*p*-Value < 5 × 10^–8^ and exclusion of GVs associated with potential confounders	1,109	IVW (random)	1.07	1.03	1.12	0.002	1,264.971	0.001	–	–	
		1,109	MR-Egger	1.05	0.97	1.13	0.187	1,264.660	0.001	2.28E-05	0.602	
Men	*p*-Value < 5 × 10-8	491	IVW (random)	1.06	0.99	1.13	0.079	515.432	0.197	–	–	0.463
		491	MR-Egger	1.11	0.998	1.22	0.055	514.180	0.199	-8.91E-05	0.276	0.381
	*p*-Value < 5 × 10^–8^ and exclusion of GVs associated with potential confounders	455	IVW (random)	1.07	0.998	1.14	0.058	484.072	0.151	–	–	0.415
		455	MR-Egger	1.14	1.02	1.26	0.026	482.058	0.159	-0.0001	0.169	0.435
Women	*p*-Value < 5 × 10^–8^	499	IVW (random)	1.07	1.01	1.13	0.022	620.752	0.0001	–	–	
		499	MR-Egger	1.02	0.91	1.12	0.731	619.030	0.0001	0.0001	0.240	
	*p*-Value < 5 × 10^–8^ and exclusion of GVs associated with potential confounders	466	IVW (random)	1.08	1.02	1.14	0.010	536.839	0.011	–	–	
		466	MR-Egger	1.04	0.93	1.15	0.448	535.993	0.011	8.38E-05	0.393	

*BMR, basal metabolic rate; CI, confidence interval; GV, genetic variant; IGF1, insulin-like growth factor 1; IVW, inverse-variance weighted; LL, lower limit; MR, Mendelian randomization; SE, standard error; UL, upper limit; WM, weighted median.*

### Genetic Associations of Basal Metabolic Rate With Neoplasm

BMR was positively associated with neoplasm overall, in men, and in women, with similar estimates (Table 3). Other MR methods and inclusion/exclusion of GVs associated with potential confounders showed directionally similar results. No MR-Egger intercept showed evidence of directional pleiotropy. BMR was positively associated with overall neoplasm using the contamination mixture method (odds ratio, 1.12; 95% confidence interval, 1.06 to 1.18). Exclusion of GVs associated with potential confounders showed directionally similar results.

**TABLE 3:** Univariable Mendelian randomization association of basal metabolic rate with neoplasm.

**Sex**	**Selection of GVs**	**No. of GVs**	**MR method**	**Odd ratio**	**95% CI LL**	**95% CI UL**	***p*-Value**	**Cochran’s *Q***		**MR-Egger**			**Outlier detected by MR-PRESSO**	***p*-Value for sex difference**
								**Statistic**	***p*-Value**	**Intercept**	**Intercept *p*-value**	***I*^2^_GX_**		
Overall	*p*-Value < 5 × 10^–8^	782	IVW (random)	1.11	1.06	1.15	2.12E-06	1,244.057	8.52E-24	–	–	–	–	
		782	WM	1.10	1.04	1.16	0.001	–	–	–	–	–	–	
		782	MR-Egger	1.13	1.01	1.25	0.036	1,243.909	6.92E-24	-3.91E-05	0.761	0.834	–	
		779	MR-PRESSO (corrected)	1.10	1.05	1.14	6.18E-06	–	–	–	–	–	rs28722029, rs78378222, rs8007058	
	*p*-Value < 5 × 10^–8^ and exclusion of GVs associated with potential confounders	750	IVW (random)	1.10	1.06	1.15	1.29E-05	1,193.855	5.77E-23	–	–	–	–	
		750	WM	1.10	1.03	1.16	0.002	–	–	–	–	–	–	
		750	MR-Egger	1.11	0.99	1.24	0.082	1,193.825	4.58E-23	-1.86E-05	0.891	0.795	–	
		748	MR-PRESSO (corrected)	1.09	1.05	1.14	2.91E-05	–	–	–	–	–	rs28722029, rs78378222	
Men	*p*-Value < 5 × 10-8	301	IVW (random)	1.07	1.03	1.12	0.002	392.156	2.69E-04	–	–	–	–	0.888
		301	WM	1.06	0.99	1.12	0.078	–	–	–	–	–	–	0.786
		301	MR-Egger	1.19	1.06	1.32	0.005	387.885	3.99E-04	-0.001	0.070	0.763	–	0.659
		300	MR-PRESSO (corrected)	1.06	1.02	1.10	0.005	–	–	–	–	–	rs78378222	0.652
	*p*-Value < 5 × 10^–8^ and exclusion of GVs associated with potential confounders	281	IVW (random)	1.07	1.02	1.12	0.008	367.939	3.21E-04	–	–	–	–	0.703
		281	WM	1.04	0.98	1.11	0.193	–	–	–	–	–	–	0.765
		281	MR-Egger	1.17	1.03	1.32	0.017	364.788	4.16E-04	-0.0005	0.121	0.693	–	0.940
		280	MR-PRESSO (corrected)	1.05	1.01	1.10	0.023	–	–	–	–	–	rs78378222	0.460
Women	*p*-Value < 5 × 10^–8^	304	IVW (random)	1.07	1.02	1.12	0.006	488.606	7.02E-11	–	–	–	–	
		304	WM	1.04	0.98	1.10	0.234	–	–	–	–	–	–	
		304	MR-Egger	1.12	0.98	1.27	0.091	487.642	6.63E-11	-0.0002	0.440	0.762	–	
		301	MR-PRESSO (corrected)	1.07	1.02	1.11	0.003	–	–	–	–	–	rs28722029, rs41277471, rs78378222	
	*p*-Value < 5 × 10^–8^ and exclusion of GVs associated with potential confounders	280	IVW (random)	1.07	1.02	1.13	0.008	454.157	1.59E-10	–	–	–	–	
		280	WM	1.02	0.96	1.09	0.453	–	–	–	–	–	–	
		280	MR-Egger	1.14	0.99	1.31	0.076	452.754	1.64E-10	-0.0003	0.353	0.686	–	
		277	MR-PRESSO (corrected)	1.07	1.02	1.12	0.004	–	–	–	–	–	rs28722029, rs41277471, rs78378222	

*BMR, basal metabolic rate; CI, confidence interval; GV, genetic variant; IVW, inverse-variance weighted; LL, lower limit; MR, Mendelian randomization; MR-PRESSO, Mendelian randomization-pleiotropy residual sum and outlier; SE, standard error; UL, upper limit; WM, weighted median.*

In multivariable MR, BMR was consistently positively associated with neoplasm after adjusting for IGF1 overall, in men, and in women (Table 4). MR-Egger and inclusion/exclusion of GVs associated with potential confounders showed directionally similar results. No MR-Egger intercept showed evidence of directional pleiotropy.

**TABLE 4:** Multivariable Mendelian randomization association of basal metabolic rate with neoplasm adjusted for insulin-like growth factor 1.

**Sex**	**Selection of GVs**	**No. of GVs**	**MR method**	**Odds ratio**	**95% CI LL**	**95% CI UL**	***p*-Value**	**Cochran’s *Q***		**MR-Egger**		***p*-Value for sex difference**
								**Statistic**	***p*-Value**	**Intercept**	**Intercept *p*-Value**	
Overall	*p*-Value < 5 × 10^–8^	1,152	IVW (random)	1.12	1.07	1.16	4.46E-07	1,776.822	1.03E-29	’	’	
		1,152	MR-Egger	1.10	1.02	1.18	0.014	1,776.399	8.90E-30	3.74E-05	0.601	
	*p*-Value < 5 × 10^–8^ and exclusion of GVs associated with potential confounders	1,109	IVW (random)	1.11	1.06	1.16	2.39E-06	1,676.315	4.30E-26	–	–	
		1,109	MR-Egger	1.10	1.02	1.18	0.018	1,676.121	3.61E-26	2.63E-05	0.720	
Men	*p*-Value < 5 × 10^–8^	491	IVW (random)	1.07	1.02	1.12	0.004	642.489	3.52E-06	–	–	0.285
		491	MR-Egger	1.11	1.03	1.20	0.006	640.241	4.04E-06	-0.0002	0.191	0.577
	*p*-Value < 5 × 10^–8^ and exclusion of GVs associated with potential confounders	455	IVW (random)	1.06	1.01	1.11	0.014	591.526	1.20E-05	–	–	0.173
		455	MR-Egger	1.10	1.01	1.19	0.021	589.997	1.26E-05	-0.0002	0.279	0.944
Women	*p*-Value < 5 × 10^–8^	499	IVW (random)	1.10	1.05	1.15	0.0001	773.460	2.23E-14	–	–	
		499	MR-Egger	1.07	0.98	1.15	0.117	772.476	2.13E-14	0.0001	0.427	
	*p*-Value < 5 × 10^–8^ and exclusion of GVs associated with potential confounders	466	IVW (random)	1.10	1.05	1.15	0.0001	693.065	2.51E-11	–	–	
		466	MR-Egger	1.09	1.004	1.19	0.041	692.992	2.07E-11	3.46E-05	0.825	

*BMR, basal metabolic rate; CI, confidence interval; GV, genetic variant; IGF1, insulin-like growth factor 1; IVW, inverse-variance weighted; LL, lower limit; MR, Mendelian randomization; SE, standard error; UL, upper limit; WM, weighted median.*

### Genetic Associations of Basal Metabolic Rate With Site-Specific Cancers

Among the 13 cancer sites in the UK Biobank, BMR was positively associated with cancer of urinary organs, cancer of respiratory system and intrathoracic organs, cancer of primary lymphoid and hematopoietic tissue, and cancer of genital organs in women (Supplementary Table 8). For all these four cancer types, multivariable MR adjusted for IGF1 showed consistent results (Supplementary Table 9).

BMR was not associated with breast cancer in BCAC or with prostate cancer in PRACTICAL Consortium (Supplementary Table 8). For all these two cancer types, multivariable MR analysis adjusted for IGF1 showed consistent results (Supplementary Table 9).

## Discussion

BMR was positively associated with cancer, consistent with theoretical expectations and previous observational and MR studies, which also suggested BMR as a risk factor or a causal risk factor of some cancers (Cornish et al., 2020; Kliemann et al., 2020). We also showed that BMR was related to benign tumors, and these associations were independent of IGF1.

### Mendelian Randomization Assumptions

A valid MR relies on three key assumptions. First, GVs must be strongly associated with the exposure. We chose GVs associated with BMR at genome-wide association significance. The F-statistic of all GVs was >10, which provides assurance that a weak instrument is unlikely (Bowden et al., 2016b). Using genetic instruments selecting using a lower *p*-value cutoff (1 × 10^–5^) gave similar results (data not shown). Second, the GVs must not be associated with the confounders of the exposure-outcome association. Though this assumption cannot be tested completely, we examined the association between the GVs and some of the important potential confounders including lifestyle and socioeconomic status. Sensitivity analysis through excluding GVs associated with these potential confounders gave mostly consistent results. Third, GVs must only exert its effect on the outcome through affecting the exposure but not *via* other pathways, i.e., no horizontal pleiotropy and no selection bias. We used MR-PRESSO to detect and exclude GVs that exhibited directional pleiotropy. Since IGF1 and BMR are highly correlated exposures, where IGF1 can affect BMR (Swanson and Dantzer, 2014), we conducted multivariable MR to take into account the effect of GVs on cancer *via* IGF1 by adjusting for IGF1 (Burgess and Thompson, 2015; Sanderson et al., 2019), and the results were consistent. We cannot exclude the possibility that for some cancers, inevitable selection of survivors of BMR, the cancer in question, and other common causes of the survival and the cancer in question may bias the estimates (Schooling et al., 2021).

### Limitations of Study

Some limitations in the current MR study are worth mentioning. First, we conducted the two-sample MR analysis using the same sample from the UK Biobank for exposures and outcomes. In the presence of weak instruments, overlapping samples in two-sample MR could result in bias toward the observational association (Burgess et al., 2016). However, the bias is proportional to the inverse of the F-statistics (Burgess et al., 2016), which were all > 10. Second, population stratification might have introduced confounding due to heterogeneity in allele frequencies between populations. However, the underlying population of the current MR study was restricted to individuals of white British ancestry, and the underlying studies address population stratification by principal component analysis. Third, cancer can be lethal so the study is open to selection bias from the inevitable selection only of survivors. Selecting only survivors of BMR and a specific cancer might attenuate or reverse estimates for cancers that are lethal at young ages, such as breast cancer, where a substantial proportion of breast cancer and breast cancer deaths occur at the age of 25 to 49 years in women in the UK (Cancer Research UK, 2021a), which could explain the null association of BMR with breast cancer in the UK Biobank, with an average age at recruitment of approximately 57 years (UK Biobank, 2020), and the null association in BCAC, where the women may be even older at recruitment (Zhang et al., 2020) as reflected by smaller estimates. A recent MR study has shown that hyperthyroidism, which raises BMR (Mullur et al., 2014), increased the risk of breast cancer, with magnitude also greater in the UK Biobank than BCAC (Yuan et al., 2020). Selecting only survivors of BMR and a competing risk of a specific cancer which might attenuate or reverse estimates for cancers that entailing survival to older ages, such as prostate cancer (Cancer Research UK, 2021b), where we observed a null association of BMR with prostate cancer in the UK Biobank and an insignificant inverse association in the PRACTICAL Consortium. The inclusion of a secondary outcome, neoplasm, which includes the less-lethal benign tumor, could serve as a validation to the results. As expected, the estimates were larger for the association of BMR with neoplasm than that for cancer using different MR methods. The larger number of cases of neoplasm (> 70,000) also allowed more consistent associations of BMR with neoplasm. Fourth, canalization, which refers to the adaptive change in genetics due to the disease, might have occurred. However, it generally biases estimates toward null (Zheng et al., 2017) and evaluation of such impact is currently not possible. Fifth, reverse causation, i.e., cancer increases BMR, may be the case. However, the mass of a cancer is unlikely to be large enough to influence BMR (Nguyen et al., 2016). Sixth, we assumed a linear association between BMR and cancer. Using summary data only, we were unable to assess any non-linear association.

### Possible Biological Mechanisms

Faster metabolism has been suggested to play an important role in cancer development. During cell metabolism, reactive oxygen species are generated as a toxic by-product (Ruggiero et al., 2008; Maciak and Michalak, 2015), which activates mutational process when in excess. More active metabolism *per se* increases the rate of genetic mutations (Maciak and Michalak, 2015) and the chance of tumor formation (Boddy et al., 2015). Cancer risk may increase when these overwhelming mutational events cannot be hedged by somatic maintenance such as DNA repair (Aktipis and Nesse, 2013; Boddy et al., 2015; Maciak and Michalak, 2015) and tissue repair (Aktipis and Nesse, 2013). Higher BMR may also compromise immunity (Maciak and Michalak, 2015), which has also been suggested to link with higher cancer risk (Aktipis and Nesse, 2013; Boddy et al., 2015; Maciak and Michalak, 2015).

### Clinical Relevance

A number of factors determine BMR, including age (Ruggiero et al., 2008; Sanchez Lopez de Nava and Raja, 2020), sex (Ruggiero et al., 2008; Jagim et al., 2019; Sanchez Lopez de Nava and Raja, 2020), ethnicity (Sanchez Lopez de Nava and Raja, 2020), lifestyle attributes like physical exercise and diet (Sanchez Lopez de Nava and Raja, 2020), and diseases like cancer (Sanchez Lopez de Nava and Raja, 2020). BMR may also be affected by body composition (Jagim et al., 2019). BMR is higher at younger ages (Ruggiero et al., 2008), in men (Ruggiero et al., 2008; Jagim et al., 2019), and in individuals with more lean body mass (Ruggiero et al., 2008; Jagim et al., 2019), similar to IGF1 (Murphy et al., 2020a). Physical activity is considered beneficial in protecting against cancer (Lugo et al., 2019). An MR study has also shown that accelerometer-measured physical activity was inversely associated with breast cancer and colorectal cancer (Papadimitriou et al., 2020). However, not all forms of physical activity affect BMR. A systematic review and meta-analysis has reported that resistant training but not aerobic exercise increases BMR (MacKenzie-Shalders et al., 2020). In rats, resistance training increases AMP-activated protein kinase level in rats (An et al., 2016), where activation of AMP-activated protein kinase inhibits mechanistic/mammalian target of rapamycin signaling which in turns suppresses cancer cell growth (Li et al., 2015). The effect of a particular type of physical activity on cancer risk through regulation of BMR requires further investigation.

Restricting caloric intake has been proposed to reduce BMR (Mole, 1990) and the effect may be independent of reduction in IGF1 (Kazemi et al., 2020). On the other hand, high protein intake increases IGF1 (Kazemi et al., 2020). Caloric restriction is beneficial in preventing cancer occurrence (Meynet and Ricci, 2014). Higher BMR was associated with higher mortality risk in a cohort study in the United States (Ruggiero et al., 2008). These findings are in line with evolutionary biology perspective that dietary restriction of specific pattern extends longevity (Soultoukis and Partridge, 2016), possibly through prevention of cancer, a major determinant of lifespan (Boddy et al., 2015). Hyperinsulinemia has been suggested as a risk factor of cancer (Vigneri et al., 2020), partly due to mitogenic effects (Vigneri et al., 2020). Notably, insulin raises free IGF1 (Vigneri et al., 2020), which in turn increases BMR (Swanson and Dantzer, 2014).

### Conclusion

Overall, our results suggest that higher BMR might cause a higher cancer risk. Intervening on modifiable targets like diet and physical exercise may help reduce the burden of this life-threatening disease by reducing BMR.

## Data Availability Statement

Publicly available datasets were analyzed in this study. This data can be found here: Website of the Breast Cancer Association Consortium (http://bcac.ccge.medschl.cam.ac.uk/) website of the Neale Lab (http://www.nealelab.is/uk-biobank/) website of the Prostate Cancer Association Group to Investigate Cancer Associated Alterations in the Genome Consortium (http://practical.icr.ac.uk/blog/).

## Ethics Statement

The studies involving human participants were reviewed and approved by the original studies (UK Biobank and genome-wide association studies). The current Mendelian randomization study used only publicly available summary data, with no original data collected. Written informed consent for participation was not required for this study in accordance with the national legislation and the institutional requirements.

## Author Contributions

CMS: conceptualization, formal analysis, supervision, and writing – review and editing. JCMN: formal analysis, writing – original draft, and writing – review and editing. Both authors contributed to the article and approved the submitted version.

## Conflict of Interest

The authors declare that the research was conducted in the absence of any commercial or financial relationships that could be construed as a potential conflict of interest.

## Publisher’s Note

All claims expressed in this article are solely those of the authors and do not necessarily represent those of their affiliated organizations, or those of the publisher, the editors and the reviewers. Any product that may be evaluated in this article, or claim that may be made by its manufacturer, is not guaranteed or endorsed by the publisher.

## Supplementary Material

Supplementary Table 1 Study details for site-specific cancers.

Supplementary Table 2 Association of genetic variants predicting basal metabolic rate with cancer, neoplasm, and site-specific cancers.

Supplementary Table 3 F-statistics for the association of basal metabolic rate and insulin-like growth factor 1 with cancer, neoplasm, and site-specific cancers.

Supplementary Table 4 Genetic variants predicting basal metabolic rate that were associated with potential confounders at Bonferroni significance.

Supplementary Table 5 Association of genetic variants predicting insulin-like growth factor 1 with cancer, neoplasm, and site-specific cancers.

Supplementary Table 6 Genetic variants predicting insulin-like growth factor 1 that were associated with potential confounders at Bonferroni significance.

Supplementary Table 7 Conditional F-statistics for the association of basal metabolic rate and insulin-like growth factor 1 with cancer, neoplasm, and site-specific cancers.

Supplementary Table 8 Univariable Mendelian randomization association of basal metabolic rate with site-specific cancers.

Supplementary Table 9 Multivariable Mendelian randomization association of basal metabolic rate with site-specific cancers adjusted for insulin-like growth factor 1.

## Abbreviations

BCAC: Breast Cancer Association Consortium
BMR: basal metabolic rate
GV: genetic variant
GWAS: genome-wide association study
IGF1: insulin-like growth factor 1
MR: Mendelian randomization
MR-PRESSO: Mendelian randomization-pleiotropy residual sum and outlier
PRACTICAL: Prostate Cancer Association Group to Investigate Cancer Associated Alterations in the Genome.
